# Notes and News

**Published:** 1888-12-08

**Authors:** 


### Notes and News.

Collected and Communicated.

The Right Honourable Lord Charles Bruce will preside at the next meeting of The Hospitals Association; and Mr. P. Michelli will read a paper on "Hospital Extravagance and Expenditure." This is one of the "burning questions" of the period, and it is expected there will be a lively discussion. The meeting will be held in the Governors' Court at Guy's Hospital, on Wednesday, December 12th, at half-past eight p.m.

In order to assist in promoting the Lady Guide Association Miss Grace Hawthorne has kindly offered a ticket benefit at the Royal Princess's Theatre, from Monday, December 3rd till Friday 14th. All tickets for any part of the house (morning or evening performances) bought of Miss Edith A. Davis, secretary, 5, Lauderdale Road, Maida Vale, will materially assist the "L.G.A."

We have received from the secretary to the Vegetarian Society, Mr. Joseph Knight, a communication on the article, "Vegetarianism and Cancer," which appeared in The Hospital of November 24th. Mr. Knight's letter contains no new facts, and is too long for publication. We do not say that vegetarianism is unsuitable for everyone. There may be persons whose health is best on vegetable diet. What we do say is that it can be no gain to vegetarianism, or anything else, that it should be credited with virtues which it has not been proved to possess.

The fourteenth annual meeting of the Glasgow Western Infirmary governors was held last week, Bailie Hamilton in the chair. Of course the report submitted by this excellent institution was as satisfactory as ever, and was received calmly by a large gathering of distinguished gentlemen. Nine beds of the infirmary have now been endowed in perpetuity, and a balance of £900 has been carried to stock account. The in-patients have numbered 3,604 during the year, and the death-rate was 7·1 per cent. The number of patients treated at the dispensary has decreased, for what reason it was not stated.

The Victoria Jubilee ward and nurses' wing of the Hereford General Infirmary was opened by Lady Elizabeth Biddulph on November 29th, 1888, in commemoration of the Queen's Jubilee. The building consists of two storeys. The ward is on the ground floor, being 72 feet long, 27 feet wide, and 13 feet 6 inches high; is lighted by lofty windows, seven on each side, and contains 16 cots, with a nurse's room, lavatories and bath-room adjoining. On the upper floor are six cubicles, four larger rooms, with lavatories and bath-room for nurses, and an isolation ward of four cots. Children up to 12 will be admitted. The 20 cots were given by as many donors at the cost of £5 each. The expenditure on the whole building was £2,880; the work being carried out by Mr. Collins, builder, Tewkesbury, from plans prepared by Mr. Rempson, F.R.I.B.A., Hereford. The number of beds in the infirmary is now increased to 103, giving full occupation to the energetic house surgeon, Mr. H. Gilbert Nicholson.

It is always profitable to see ourselves as others see us. The success of The Hospital has been great, and it is gratifying to find the *Pall Mall Gazette* last Saturday explaining this fact to its readers in the following forcible fashion: "*Apropos* of the hubbub in medical circles, the following figures of the circulation of the three leading medical weeklies will be not without interest. They do not profess, of course, to be exact, but I have reason to believe them substantially accurate: *British Medical Journal*, 14,000 (a circulation which has doubled during the present editorship); The Hospital, 9,000; *Lancet*, 7,000."

An amusing dramatic entertainment was given at Netley Hospital, on November 21st, by various soldiers, under the direction of Sergeant-Major Vidler. There was a large audience, including Colonel Crichton, Surgeon Lumsden, the Rev. Mr. Beamish, and several of the nursing sisters. The first night the entertainment was given free to the convalescent patients, choir boys, and others connected with the hospital. The second evening a small charge was made, and the money raised was devoted to sending a child to the Army Guilds' Home for a year.

A curious scene was witnessed at St. Thomas's Hospital last week. Fifty time-expired soldiers of the Dublin Fusiliers arrived at Waterloo Station from Gosport in states of intoxication varying between helplessness and madness. About thirty were insensible, and lay on the platform, one rolled into the 6-ft. way, and had a narrow escape of being run over by a train; five were placed in a railway van and driven to St. Thomas's Hospital, where they were placed on the floor of the further casualty ward and attended by the house physicians and other members of the staff. The men were perefectly unconscious and their faces all bloated and discoloured; blood oozed from their various wounds and bruises, and the galvanic battery had to be applied in two cases before there was any sign of returning animation. A more ghastly or disgraceful scene of debauchery it is impossible to imagine, and it is to be hoped the Secretary of State for War will make full inquiries into the occurrence.

We record with deep regret the death of Major Alexander Ross, M.P., at his residence, 9, Upper Berkeley-street, Portman-square. Major Ross had been for some time out of health, and it had been decided that he should go to the South of France for the winter. Last Monday he seemed to be in much the same health as usual, and after breakfast walked to the Carlton Club. He returned home about eleven, and at one was found reclining in a chair in his sitting-room, apparently lifeless. Dr. Broadbent was at once sent for, and pronounced the major dead; death having resulted from heart disease. Major Ross, who sat in Parliament for the borough of Maidstone, was a Conservative in politics and a steady supporter of the Union as it now exists. In addition to his parliamentary duties he took a prominent part in hospital work and other public charities. He was Chairman of the Middlesex Hospital, and gave the duties of his office constant and intelligent attention. He was also Chairman of Council of The Hospitals Association, with whose objects and work he was in the fullest sympathy. Major Ross was born on October 21st, 1829. He was the son of the late Mr. Charles Ross, M.P., and Lady Mary Cornwallis, daughter of the Marquis Cornwallis. He was educated at Eton and Christ Church, Oxford, and graduated B.A. in 1851, and M.A. in 1854. He served twenty years in the West Kent Militia, from which he retired as Major. He was a Justice of the Peace for Middlesex and a member of the Metropolitan Asylums Board. Great sympathy is felt in Maidstone with the members of Major Ross' family, many of whom reside in the borough.

The following vacancies are announced this week, the amount of salary in each case being given after the name of the institution :—

**Resident Medical Officers.**

Royal Albert Hospital, Devonport. Assistant House Surgeon.—Board, etc.
North Eastern Hospital for Children. Junior House Surgeon.—£30.
Sunderland Infirmary. House Physician.—£80, Board, etc.
Nottingham General Hospital. Medical Assistant.—Board, etc.
Nottingham General Hospital. Surgical Assistant.—Board, etc.
Wolverhampton General Hospital. Resident Assistant. - Board, etc.
Throat Hospital, Great Portland Street. House Surgeon.—Board, etc.

**Non-resident Medical Officers.**

London Temperance Hospital. Registrar and Chloroformist.—£50.
University of Edinburgh. Examiner in Medical Jurisprudence. £75.
Westminster Hospital. Curator of Museum.—£40.

---

The quarterly meeting of the governors of the Hospital for Consumption, Brompton, was held on November 29th, Mr. Beckwith in the chair. Amongst those present were : Sir W. R. Brown, Dr. Tatham, the Rev. F. E. C. Byng, and others. The whole of the 321 beds of the institution are now occupied, and 582 patients have been admitted since July, 402 have been discharged, many greatly relieved, and 90 have died. The weekly entertainments which have been so kindly given by various ladies and gentlemen for many winters, have recommenced, and prove of the greatest benefit and interest to the patients.

---

According to the annual report of the St. John's Ambulance Association just published, 596 "Detached classes" have been held during the year, and the certificates awarded have amounted to 22,181, as compared with 17,718 the previous session, making a total of nearly 150,000 certificated pupils. Classes for police, railway employés, firemen, and working people generally have largely increased, especially in the colliery and mining districts, where a great impetus has been given to the sale of ambulance material by the Miners' Regulation Act, which came into force in January last, and which compels owners to provide stretchers and other appliances. The Prince of Wales has accepted the office of president of the Association.

---

The Silver Fête at the Dublin Children's Hospital has been a great success. Among the many distinguished patrons of the event were the Duke and Duchess of Connaught, the Marchioness of Londonderry, and Princeess Edward of Saxe-Weimer. Sister Bessie, the lady superintendent of the hospital, and several other Red Cross sisters were present in their picturesque garb, and presided over one of the stalls. In one of the wards the small patients had a dolls' stall all of their own ; and, needless to say, attracted many buyers. The whole of the building devoted to the fête was draped in silver paper, and silver banners and decorations of every sort were called into request. The effect was very good. Princess Edward of Saxe-Weimar spent a whole afternoon within the precincts of the building, Mr. Kempe being in attendance.

---

The Queen of Portugal lately visited M. Pasteur's Laboratory, which is about to be vacated for the Pasteur Institute in Rue Dutot, Paris. She was present at the inoculation of the numerous patients, of whom there were about one hundred and fifty. The royal party then drove to the new institute, which was formally opened on November 14th. It comprises two vast buildings, one fronting Rue Dutot, the other Rue des Fourneaux. They are linked together by a vast gallery, which could, if needed, serve as a lecture-room. Part of the building is set aside for the study of microbes in general, and there are numerous laboratories, inoculation-rooms, &c. During the month of October only two dogs were certified to have died of rabies in London, though ten were killed in the streets, and over a thousand taken to the Dogs' Homè.

An amusing story comes from Paris about a wealthy client of a poor dentist. The client had toothache, and wanted the tooth taken out, but refused chloroform. The dentist proceeded to produce a large array of hideous instruments and made all sorts of ghastly preparations, till he frightened his patient into consenting to take an anæsthetic. When the client slowly recovered consciousness the tooth was still there and still aching, and he had a headache to boot ; but the dentist had relieved him of one source of ill—he had gone off with his client's purse containing much filthy lucre !

---

The University College Hospital is issuing this year a special Christmas appeal. The managers call attention to the facts that the hospital is both a large general hospital and a series of special hospitals, it being provided with a separate department for every important medical and surgical specialty. It is situated in a densely-populated neighbourhood, and immediately contiguous to the London and North-Western, the Midland, and the Great Northern Railway termini. It is dependent entirely upon voluntary contributions, and is at the present time urgently in want of funds. The hospital receives annually nearly 3,000 in-patients, and over 34,000 out-patients. The ordinary annual expenses are £21,000, whilst the reliable income is only £6,000. There is a scheme on foot to rebuild the hospital, and for this object donations are earnestly solicited. The hospital is practically *free*, no patient being turned away if there is a vacant bed.

---

Miss Chreiman's lecture on the "Physical Culture of Women," improves as time goes on. It has been delivered before the Society of Arts, the Parkes Museum, and numerous suburban audiences, and Miss Chreiman has the tact to adapt and alter it on every occasion. In speaking before the Richmond Athenæum lately, Miss Chreiman fought shy of the subject of corsets, which is usually fruitful of much discussion, but the chairman and doctors present were not to be debarred from thrashing bare the old subject. This was a practical point they felt, whereas Miss Chreiman's remarks about a good birth and orderly growth dealt with large generalisations. After listening calmly to all the strictures of her critics, Miss Chreiman made a spirited reply, in which she stated that she had purposely avoided talking to such an intelligent audience on such stale subjects as stays and high heels, and had rather tried to bring before them a type of a truly wide and beneficial physical culture, in which such subjects could have no possible place. We think Miss Chreiman had the best of the argument.

---

It seems that the objectionable practice of making the medical officer of the infirmary supply drugs and appliances out of his salary still exists at Eastbourne, and has lately been the subject of much discussion. The result of this method of payment is that the more patients the doctor has the less remuneration he receives—an absurd state of affairs —and the temptation to provide inferior medicines and ill-adapted appliances is, of course, very great. The medical officer at Eastbourne Union Infirmary receives £100 per annum, and during the last few years there have been a good many operations which required not only a consumption of material at the time, but the supply of dressings for weeks after. Now there is a new infirmary, the guardians might have the sense to alter this wretched arrangement without so much wrangling and discourtesy as seems to prevail at their meetings. Why is it that the discussion of guardians in sitting usually reads like this ?—

*A.*—"I hope the Board will not wander from my resolution."
*B.*—"Excuse me. Am I allowed to speak, or not ?"
*A.*—"My motion has nothing to do with the question of salary."
*B.*—"I know what I am talking about." And so on.

